# Transcriptome analysis of the fungal pathogen *Fusarium oxysporum* f. sp. *medicaginis* during colonisation of resistant and susceptible *Medicago truncatula* hosts identifies differential pathogenicity profiles and novel candidate effectors

**DOI:** 10.1186/s12864-016-3192-2

**Published:** 2016-11-03

**Authors:** Louise F. Thatcher, Angela H. Williams, Gagan Garg, Sally-Anne G. Buck, Karam B. Singh

**Affiliations:** 1 CSIRO Agriculture and Food, Centre for Environment and Life Sciences, Wembley, Western Australia 6913 Australia; 2 The Institute of Agriculture, The University of Western Australia, 35 Stirling Highway, Crawley, Western Australia 6009 Australia

**Keywords:** *Fusarium* wilt, Vascular wilt, RNA-Seq, DEG, root pathogen, Necrotroph, Hemibiotroph, Effector, Secreted In Xylem, Small secreted protein

## Abstract

**Background:**

Pathogenic members of the *Fusarium oxysporum* species complex are responsible for vascular wilt disease on many important crops including legumes, where they can be one of the most destructive disease causing necrotrophic fungi. We previously developed a model legume-infecting pathosystem based on the reference legume *Medicago truncatula* and a pathogenic *F. oxysporum* forma specialis (f. sp.) *medicaginis* (*Fom*). To dissect the molecular pathogenicity arsenal used by this root-infecting pathogen, we sequenced its transcriptome during infection of a susceptible and resistant host accession.

**Results:**

High coverage RNA-Seq of *Fom* infected root samples harvested from susceptible (DZA315) or resistant (A17) *M. truncatula* seedlings at early or later stages of infection (2 or 7 days post infection (dpi)) and from vegetative (in vitro) samples facilitated the identification of unique and overlapping sets of *in planta* differentially expressed genes. This included enrichment, particularly in DZA315 *in planta* up-regulated datasets, for proteins associated with sugar, protein and plant cell wall metabolism, membrane transport, nutrient uptake and oxidative processes. Genes encoding effector-like proteins were identified, including homologues of the *F. oxysporum* f. sp. *lycopersici* Secreted In Xylem (SIX) proteins, and several novel candidate effectors based on predicted secretion, small protein size and high *in-planta* induced expression. The majority of the effector candidates contain no known protein domains but do share high similarity to predicted proteins predominantly from other *F. oxysporum* ff. spp. as well as other *Fusaria* (*F. solani, F. fujikori, F. verticilloides, F. graminearum* and *F. pseudograminearum*), and from another wilt pathogen of the same class, a *Verticillium* species. Overall, this suggests these novel effector candidates may play important roles in *Fusaria* and wilt pathogen virulence.

**Conclusion:**

Combining high coverage *in planta* RNA-Seq with knowledge of fungal pathogenicity protein features facilitated the identification of differentially expressed pathogenicity associated genes and novel effector candidates expressed during infection of a resistant or susceptible *M. truncatula* host. The knowledge from this first in depth *in planta* transcriptome sequencing of any *F. oxysporum* ff. spp. pathogenic on legumes will facilitate the dissection of *Fusarium* wilt pathogenicity mechanisms on many important legume crops.

**Electronic supplementary material:**

The online version of this article (doi:10.1186/s12864-016-3192-2) contains supplementary material, which is available to authorized users.

## Background

*Fusarium oxysporum* is a soil-borne fungal pathogen capable of causing widespread destructive losses on over 100 different plant species. Specialised pathogenic strains of this root-infecting fungus are classified into host-specific sub-species known as *formae speciales* (ff. spp.) (singular *forma specialis*, abbreviated: f. sp.) based on the host species they cause disease on, and are responsible for the disease known as *Fusarium* or vascular wilt [1–4]. The spores of this pathogen can survive in soil for decades, thus it is particularly difficult to eradicate following soil contamination [1]. Important agronomical crops which are affected by *Fusarium* wilt include cotton (*Gossypium* species), horticultural crops such as bananas (*Musa* species), cucurbits/melons (*Cucurbitaceae* species), strawberries (*Fragaria* × *ananassa*), lettuce (*Lactuca sativa*) and tomatoes (*Solanum lycopersicum*), and many grain and pasture legume species such as chickpea (*Cicer arietinum*), common bean (*Phaseolus vulgaris*), field pea (*Pisum sativum*), lentil (*Lens culinaris*) and lucerne/alfalfa (*Medicago sativa*) [1–6].

It was proposed for some isolates of a *F. oxysporum* f. sp. that their ability to cause disease on specific hosts arose through descent from a monophyletic origin. However, for others it was proposed their genetic heterogeneity was polyphyletic in origin and for several this has now been experimentally demonstrated [3, 7–11]. Comparative genomic studies across *Fusaria* identified ‘core’ chromosomes containing genes required for vegetative growth and metabolism, and ‘non-core’ chromosomes (or parts of) and gene content geared towards pathogenicity [9, 12]. The latter, also referred to as lineage-specific, accessory or conditionally dispensable chromosomes (CDCs), lack house-keeping genes and are poorly conserved across other *Fusaria* or other fungi, but may show higher levels of disparate conservation amongst specific *F. oxysporum* ff. spp. [5, 9, 13]. In a series of elegant experiments, it was demonstrated that the small CDC 14 (~1.6 Mb) and parts of other CDCs from some *F. oxysporum* f. sp. *lycopersici* (*Fol*) isolates (including the reference isolate *Fol-*4287) could be horizontally transferred to other isolates, enabling the transfer of pathogenicity [9, 10]. Conversely, a loss of pathogenicity or virulence can also result from the loss of all or parts of *Fol* Chr14 [10, 14–16]. The small *Fol*-4287 CDC 14 is referred to as the *Fol* ‘pathogenicity’ chromosome as it contains the majority of known *Fol in planta* expressed effectors, some of which have been shown to interfere with the host’s resistance response and/or are required for virulence [3, 9, 13, 14, 17, 18].

The CD chromosomes or scaffolds of *F. oxysporum* ff. spp. analysed in some detail to date (f. sp. *lycopersici, melonis, medicaginis, ciceris, pisi, cubense*) are enriched in repetitive elements with CDC encoded transposable elements (TEs) accounting for nearly 75 % of all TEs in the *Fol*-4287 genome [5, 9, 13, 19]. While only 20 % of *Fol*-4287 genes on these chromosomes can currently be functionally classified based on the presence of conserved domains, they are enriched for genes related to pathogenicity such as known and putative effectors, fungal transcription factors and genes with roles in signal transduction and secondary metabolism [9]. Similarly, over half of the predicted proteins from the legume-infecting ff. spp. *medicaginis* (*Fom*-5190a), *ciceris* (*Foc*-38-1) or *pisi* (*Fop*-37622) genome assemblies assigned to predicted dispensable scaffolds are unclassified proteins with no known function and those that could be assigned functional annotations grouped into similar categories as those enriched on *Fol* CDCs [5].

*F. oxysporum* is one of the major pathogens of legumes, particularly chickpea, the second most important global grain legume crop (FAO: www.fao.org). Typical annual yield losses due to pathogenic isolates of this host, *F. oxysporum* f. sp. *ciceris,* are upwards of 10 % but under favourable disease conditions yield loss can reach 100 % [20–23]. With the majority of the world’s chickpea production originating from one country (India) (FAO: www.fao.org), disease outbreaks and a lack of control mechanisms can have severe impact on global chickpea supplies. Various sources of host resistance in chickpeas and other legumes have been identified, but the underlying genetic or molecular mechanisms (e.g. *Resistance* or *Pathogenesis-Related* genes, signalling pathways) are yet to be fully elucidated [3, 20, 24, 25]. Parallel to this, genetic and molecular mechanisms responsible for individual *F. oxysporum* f. sp. pathogenicity on legumes is poorly understood. Towards the aim of transferring knowledge to complex legume species, pathosystems utilising the reference legume *Medicago truncatula* have been developed to dissect the interaction between *F. oxysporum* and legume hosts [5, 26–28]. Utilising the pathogenic *F. oxysporum* f. sp. *medicaginis* strain *Fom*-5190a (herein referred to as *Fom*) isolated from alfalfa (Lucerne, *M. sativa*) we developed a robust *Medicago-F. oxysporum* pathosystem and identified a resistant and highly susceptible accession [5, 29]. These are the reference *M. truncatula* accession A17 (resistant) and the accession DZA315 (susceptible).

Comparative genomics of a comprehensive draft *Fom* genome assembly against the genome assemblies of legume-infecting and other host pathogenic *F. oxysporum* ff. spp. as well as other *Fusarium spp.* and fungal plant pathogens, identified pathogenicity related gene content possibly geared towards legume host-specificity [5]. This information coupled with RNA-Seq data from an early time-point during infection of the susceptible *M. truncatula* accession (DZA315), facilitated the shortlisting of a set of 10 *Fom* key effector candidates. Herein, we set out to expand on this analysis and identify potential differences in the expressed pathogenicity profile of *Fom* during early infection of susceptible DZA315 versus resistant A17 *M. truncatula* hosts. One of the limiting factors in early detection of fungal transcripts in root colonised tissues is their poor relative abundance compared to host transcripts and to overcome this, high coverage sequencing or specific sequence capture of fungal transcripts is necessary. We overcame this constraint by conducting high coverage RNA-Seq and herein present one of the most comprehensive early *in planta* expressed *F. oxysporum* transcriptomes, and the first for a legume infecting formae speciales to our knowledge. Our RNA-Seq involved analysis of root samples collected at the early time-point of 48 h after infection and at a later time-point of 7 days when disease symptoms were starting to manifest in the susceptible plants. At the early time-point, although the degree of root colonisation between resistant and susceptible plants was alike, the number, level of induction, and composition of *in planta* expressed *Fom* genes was higher and more diverse in the susceptible interaction. By 7 days post inoculation a significant increase in colonisation of susceptible plants was evident coupled with increased expression of genes predicted as effectors or associated with protein, sugar and plant cell wall breakdown, membrane transport and nutrient uptake. We discuss these differences and the discovery of new candidate *F. oxysporum* effectors.

## Results

### Quantification of *Fusarium* growth in resistant and susceptible *M. truncatula* accessions

**Fig. 1:**
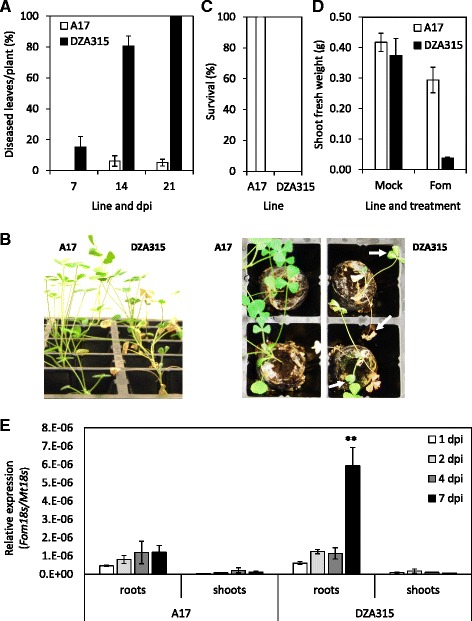
Disease symptoms and *Fom* colonisation of resistant and susceptible *M. truncatula* accessions. **a**-**d** Disease symptoms of *Fom* inoculated A17 and DZA315 *M. truncatula* seedlings. **a** Percentage of diseased seedlings at 7, 14 and 21 days post inoculation (dpi) with **b**) an image of representative seedlings at 14 dpi. White arrows highlight disease symptoms of wilting, chlorotic and necrotic leaves. **c** Average survival and **d** above ground fresh weight of mock (control) or *Fom* inoculated seedlings at 21 dpi. For **a**, **c** and **d** values are averages ± SE (n = 10). Similar results were obtained in independent experiments. **e** Relative *Fom* fungal abundance determined by qRT-PCR analysis of *Fom*_*18S* relative to *M. truncatula_18S* in samples harvested at 1, 2, 4 and 7 dpi. Samples are averages ± SE of 4 biological replicates consisting of pools of 10 seedlings. Asterisks indicate values that are significantly different (**P < 0.01 Student’s *t*-test) between A17 and DZA315 at the respective time point

We previously identified the reference *M. truncatula* accession A17 to display moderate to strong resistance to *Fom* [29] whilst the DZA315 accession was susceptible [5]. To examine differences in disease progression between these two hosts we infected both alongside each other and quantified *Fom* growth in root and shoot tissues over an infection time-course (Fig. 1). Within 14 days post inoculation (dpi) 97 % of DZA315 plants had visible disease symptoms of wilting, and chlorotic or necrotic leaves, while only 30 % of A17 plants were diseased and of these, only 6 % of their leaves on average had visible disease symptoms (Fig. 1a, b). By 21 dpi all A17 plants survived, although the number of diseased plants had risen to 40 %, these again displayed limited symptoms (on average 5 % of leaves were diseased per plant, Fig. 1a). In contrast, all DZA315 plants were dead by 21 dpi (Fig. 1c). At 21 dpi the *Fom* inoculated A17 seedlings were visibly smaller than mock or control inoculated seedlings, showing a 30 % reduction in shoot fresh weight (Fig. 1d). The limited disease progression observed in A17 suggests *Fom* is able to colonise A17 seedlings but that the molecular resistance mechanisms employed by A17 plants to control pathogen spread results in reduced growth.

To determine the extent of *Fom* colonisation in A17 and compare this to levels in susceptible accession DZA315, we quantified the relative amount of fungal biomass in root and shoot tissues of A17 and DZA315 seedlings by qRT-PCR over the early stages of infection between 1 and 7 dpi. Both A17 and DZA315 had similar levels of *Fom* biomass at 1, 2 and 4 dpi in roots, with some growth also detected in shoots (Fig. 1e). However, by 7 dpi the relative abundance of *Fom* had risen sharply and significantly in DZA315 root tissues to levels ~5-fold greater than levels in A17 which remained similar to those detected at its 4 dpi time-point.

### In vitro and *in planta Fusarium* RNA-sequencing

We hypothesized that increased *Fom* colonisation of susceptible DZA315 seedlings may be associated with changes in its pathogenicity gene expression profile compared to colonisation of a resistant A17 host. To capture *Fom* genes expressed in resistant and susceptible plants and to compare and contrast their expression profiles we generated high coverage RNA-Seq data from infected root and shoot tissues of A17 and DZA315 seedlings at 2 dpi where we detected no difference in host colonisation levels and at the later time-point of 7 dpi when disease symptoms start to manifest in susceptible plants (Fig. 1). We confirmed that disease progressed as expected in seedlings used for RNA-sequencing by scoring the percentage of diseased plants and survival at 7-21 dpi on plants from the same experiment that were not harvested for RNA extractions (Additional file 1). We also collected data from *Fom* mycelia grown vegetatively (in vitro) in order to compare levels of induction upon host detection and use this as a feature to facilitate identification of effectors or other pathogenicity-associated genes.

Based on our previous qRT-PCR results (Fig. 1e) we estimated the relative abundance of *Fom* transcripts to *M. truncatula* transcripts in harvested root tissues would range from 0.05-0.5 %. Using this as a guide we conducted stranded RNA-Seq on three biological replicates for each treatment/tissue on an Illumina HiSeq platform (2x100 bp) generating 10.76−12.82 Gb data for each sample. After read processing (quality trimming and adaptor removal), ~55 million paired end reads were obtained for each sample and mapped to our *Fom* reference genome assembly [5] using TopHat2 [30]. For the in vitro samples 93-94 % of reads could be mapped to the *Fom* genome assembly, while for infected root samples the percentage of reads mapped ranged from 0.02-0.04 % at 2 dpi to 0.02−0.19 % at 7 dpi (Table 1). Less than 0.01 % of reads could be mapped from the shoot samples, even at the later time point of 7 dpi (data not shown). Combined, the in vitro and *in planta* RNA-Seq supported expression of 16,473 of the 16,858 predicted *Fom* gene models with 12,312 expressed *in planta* based on mapping of one or more reads in any of the *in planta* samples. Correlating with our fungal biomass results (Fig. 1e), similar percentages of reads could be mapped to the *Fom* genome in samples harvested from A17 and DZA315 roots at 2 dpi but at 7 dpi the number and percentage of mapped *Fom* reads was up to 10 times greater in the DZA315 samples compared to A17.

**Table 1:** Mapping results of RNA-Seq data from *Fusarium* infected A17 (resistant) or DZA315 (susceptible) root samples

	in vitro	Inf. A17 2 dpi	Inf. A17 7 dpi	Inf. DZA315 2 dpi	Inf. DZA315 7 dpi
Pre-processed reads for each replicate (million pairs)	58.7 ± 1.5	56.2 ± 1.4	55.5 ± 1.6	53.4 ± 0.9	55.4 ± 0.6
Range of read pair numbers mapped	54,789,566 ± 1,026,861.7	12,626.0 ± 951.5	30,343.2 ± 6,309.8	14,713.8 ± 1,745.2	91,061.5 ± 5,934.4
% range of reads mapped	92.6-94.2	0.016-0.028	0.021-0.080	0.021-0.038	0.139-0.194
Pairs with multiple alignments (%)	0.2	0.4-0.5	0.4-0.5	0.2-0.3	0.4-0.5

*Inf Fom* infected, *dpi* days post inoculation; ±: SE

### Identification of differentially expressed *Fusarium* genes

To identify genes with a high potential for involvement in pathogenicity, we set out to identify *Fom* genes differentially expressed between vegetative (in vitro) and *in planta* growth conditions with the premise that genes involved in fungal pathogenicity would be switched on or more highly and rapidly expressed upon detection of a suitable host [6, 18, 19, 31–33]. Aligned read counts (generated from the maploci and genDEseq subprocesses within the BioKanga toolkit [http://sourceforge.net/projects/biokanga/files/]) were used with a normalisation step to identify differentially expressed genes (DEGs) between each dataset (growth condition, dpi, plant accession) with EdgeR [34]. DEGs were selected based on a ≥ 2-fold change and a False Discovery Rate (FDR) ≤ 0.05. Due to low proportion of *Fom* reads in the RNA-Seq datasets, to decrease the number of false positives based on mapping of only a few reads or mapping of multiple reads to only one location, we added an additional criteria of at least 25 % read coverage of the predicted gene model in each of the 3 *in planta* replicates. In the early 2 dpi dataset replicates this equated to on average 84−92 % of transcripts with a minimum of 5 mapped reads, with individual gene models covered on average by 85 reads. Similar additional DEG criteria have been used in other lowly *in planta* expressed pathogen or endophyte studies [35, 36]. Full details of DEGs and characteristics of their encoded proteins are listed in Additional files 2 and 3.

**Fig. 2:**
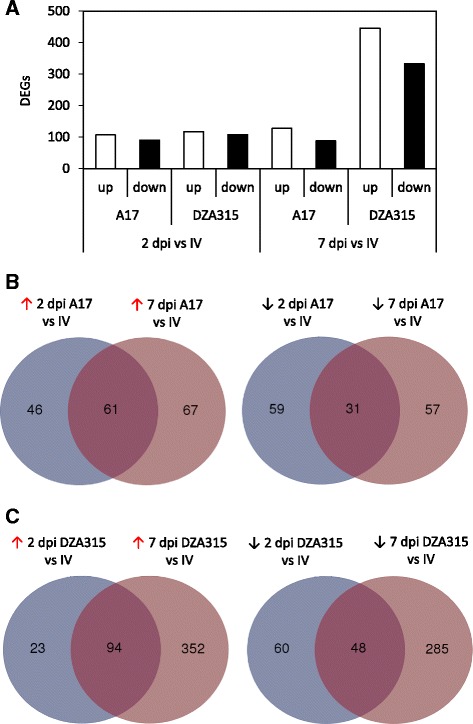
Number of *Fom* genes differentially expressed between in vitro and *in planta* samples. **a** DEGs detected between *Fom* grown in vitro (IV) and during infection of A17 or DZA315 roots at 2 or 7 days post inoculation (dpi). **b**-**c** Venn diagrams of DEGs in overlapping datasets from **b**) A17 and **c**) DZA315. Red and black arrows indicate up- or down-regulated *in planta* respectively

At both 2 and 7 dpi the number of DEGs induced/repressed *in planta* in DZA315 was greater than those detected in A17 suggesting a larger degree of transcriptional reprogramming in the susceptible plant interaction (Fig. 2, Table 2). This was particularly evident at 2 dpi even when *Fom* fungal biomass was comparable between susceptible and resistant plant roots (Fig. 1e) with 11 % more DEGs in the DZA315 up-regulated dataset compared to A17, and 30 % more genes with ≥ 95 % read coverage (Table 2). By 7 dpi the difference in number of DEGs between DZA315 and A17 increased by over 3.5-fold, likely associated with the greater fungal colonisation of DZA315 roots (Fig. 1e, Table 2). In DZA315 80 % of DEGs up-regulated at 2 dpi were also up-regulated at 7 dpi, while this was only observed for 57 % of DEGs in A17 (Figs. 2b, c). Considering the larger number of mapped reads and DEGs identified in DZA315 at 7 dpi, less overlap between the 2 and 7 dpi DZA315 datasets might be expected. This result suggests a fast, prolonged, and concerted expression of components of the *Fom* pathogenicity arsenal during infection of susceptible plants. With the exception of *Fom*_*00898* (expression down at 2 dpi DZA315 but up at 7 dpi DZA315) there were no genes detected in both up- and down-regulated datasets of either genotype. Interestingly *Fom*_*00898* encodes a protein with characteristics of a small secreted protein (SSP; protein length ≤ 300 amino acids, predicted to be secreted (SignalP) and containing ≤ one transmembrane domain in the N-terminal region [5]), contains a CFEM domain possibly associated with fungal pathogenicity [37], and is predicted as a putative effector by the fungal effector prediction software EffectorP [38].

**Table 2:** Differentially expressed *Fusarium* genes detected during infection of A17 or DZA315 roots

Proportion of gene model coverage	A17 2 dpi vs IV		A17 7 dpi vs IV		DZA315 2 dpi vs IV		DZA315 7 dpi vs IV	
	up	down	up	down	up	down	up	down
≥25 %	107	90	128	88	117	108	446	333
≥50 %	64	21	63	34	66	35	281	141
≥95 %	13	1	25	5	17	4	105	28

*IV* in vitro. Note, DEGs in ≥ 95 % are also captured within the ≥ 50 % and ≥ 25 % datasets. Likewise, DEGs in ≥ 50 % are captured within the ≥ 25 % dataset

We also examined RNA-Seq data from the same experiment, at the same level of sample read coverage obtained from the shoots of the *Fom* inoculated DZA315 or A17 plants but due to the relative low abundance of fungal transcripts in shoot tissues at our early sampling time-points only a handful of DEGs could be identified (data not presented).

### Protein characteristics of differentially expressed genes with a focus on *in planta* up-regulated datasets

To assess differences in pathogenicity profiles of *Fom* during interactions with susceptible or resistant *Medicago* hosts, we focussed the remainder of our studies on DEGs up-regulated *in planta* versus in vitro. To interpret putative functions, we interrogated their predicted protein characteristics including gene ontology (GO) terms, Pfam domains and pathogenicity associated characteristics such as encoding SSPs or similarity to known fungal effectors. Although fungal effectors generally lack similarity to other proteins, they may share common protein motifs or characteristics such as a secretion signal, small size (generally less than 300 aas), increased number of paired cysteines, proximity to repetitive DNA and these were therefore included in our analyses. *Fom* protein characteristics were previously determined by [5] and supported by annotations listed in publicly available *Fusarium* spp. genome projects, PHI-base [39, 40] and GenBank. We also incorporated an assessment of putative chromosomal location based on our previous study [5]. *Fom* scaffolds representing putative CD chromosomes were predicted based on the criteria of having no match to designated core chromosomes of *F. oxysporum* f. sp. *lycopersici* or *F. solani*, whose genome assemblies contain well characterised core and CD/accessory chromosome sequences.

One fifth of the predicted proteins in up-regulated DEG datasets could not be assigned functional annotations and were annotated as uncharacterised. This included 24 (22 % of total in the dataset) and 27 (21 %) in A17 at 2 and 7 dpi respectively. In DZA315 infected plants there were a similar number of uncharacterised proteins at 2 dpi (22 DEGs; 19 % of dataset) but within the 7 dpi dataset the total number of uncharacterised proteins more than tripled to 78 (representing 17 % of the dataset). Nearly half of the latter were predicted as secreted and as putative effectors by EffectorP.

Of proteins in the up-regulated datasets that could be assigned GO terms, 14−18 % were assigned to biological processes, 16−18 % molecular functions, and 3−4 % cellular components. Thirty-six and 33 GO terms could be assigned respectively to A17 and DZA315 2 dpi datasets, and 45 and 69 respectively in the 7 dpi datasets. Of these, metabolic and catalytic activity represented the majority of classified proteins in all datasets, followed by catabolism, binding, hydrolase activity and biosynthesis in different percentages (Additional file 4). The number of encoded proteins represented by these GO terms were 3-6 times more in the DZA315 7 dpi dataset compared to A17 at the same time-point. For example, DZA315:A17 carbohydrate metabolism 22:7, lipid metabolism 13:2, hydrolase activity 39:18, catabolism 37:10, signal transduction 5:0 (Additional file 5).

**Fig. 3:**
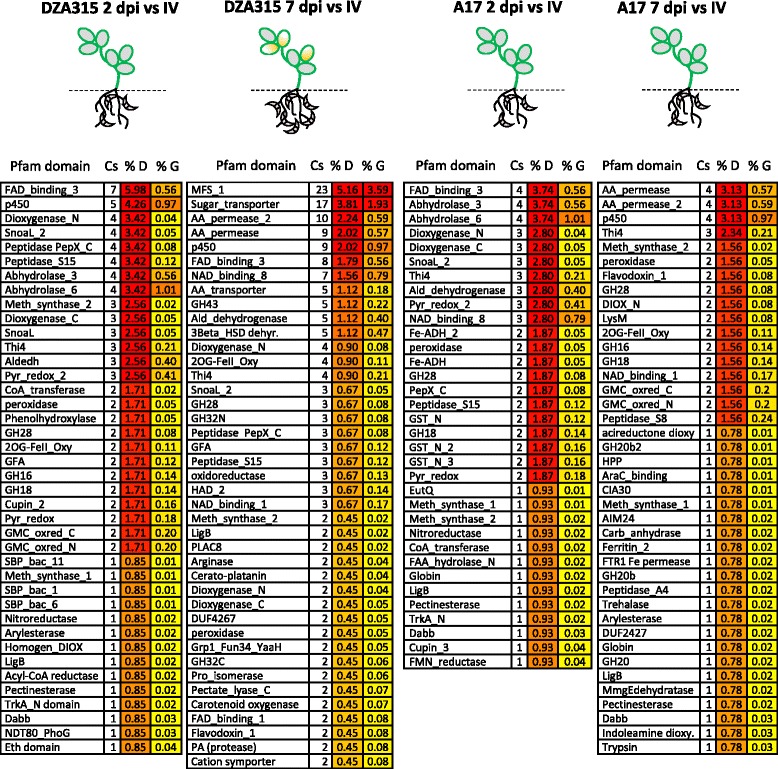
Pfam domains more abundant in the *in planta* up-regulated datasets. Pfam domains enriched in the *in planta* up-regulated datasets from resistant (A17) and susceptible (DZA315) accessions relative to in vitro growth conditions are listed. Schematic figures illustrate the tissue sampled (represented as root tissues below the dashed line infected with *Fom* (purple spores), highlighting the chlorotic leaves visible at the later stage of the susceptible interaction). Enriched Pfam domains were identified based on comparisons against the total *Fom* protein set using Fisher’s exact test with a significance threshold of *p* ≤ 0.05. Values are ranked by representation of Pfam domains with colour coding signifying increasing abundance within each dataset. Cs: counts of Pfam domain containing proteins in DEG dataset. % D: % representation of Pfam domain containing proteins in DEG dataset; % G: % representation of Pfam domain containing proteins in whole genome. Further details are provided in Additional File 6. Aldeh: Aldehyde dehydrogenase; AIM24: Mitochondrial biogenesis; CIA30: mitochondrial Complex I intermediate-associated protein; Dabb: Stress responsive A/B Barrel Domain; EutQ: Ethanolamine utilisation protein; GH: glycoside hydrolase; Grp1_Fun34_YaaH: acetate transporter; GFA: Glutathione-dependent formaldehyde-activating enzyme; GST: Glutathione S-transferase; HAD: Haloacid dehalogenase-like hydrolase; LigB: LigB subunit of aromatic ring-opening dioxygenase; Meth_synthase: methionine synthases; NDT80_PhoG: DNA binding-family; PLAC8: Placenta-specific gene 8 protein; Pyr_redox: Pyridine nucleotide-disulphide oxidoreductase (includes oxidoreductases, NADH oxidases and peroxidases); SBP: Bacterial extracellular solute-binding protein; Thiamine4: thiamine biosynthetic enzyme

For *Fom* genes up-regulated *in planta*, a significant over-representation (Fisher’s exact test *p* ≤ 0.05) of domains associated with degradation of proteins and sugars/carbohydrates (e.g. glycoside hydrolase, pectate lyase, protease), membrane transport (e.g. sugar, amino acid and Major Facilitator Superfamily (MFS) transporters, nucleobase cation symporter-1, permease) and oxidative processes (e.g. 3-beta hydroxysteroid dehydrogenase, oxidoreductase, cytochrome p450s) was observed, particularly in DZA315 (Fig. 3, Additional file 6). Encapsulated, these results suggest host cell wall and membrane degradation along with nutrient transport are initiated earlier upon infection of susceptible plants, evident as early as 2 dpi compared to the resistant host interaction. By 7 dpi these processes were even more apparent during *Fom* infection of susceptible plants, correlating with an increase in fungal biomass at this time point (Fig. 1e).

Searching for proteins involved in regulation of *Fom* pathogenicity-associated gene expression, we identified four proteins annotated as fungal transcription factors with one (*Fom*_*15733*) common to three of the datasets (2 dpi A17, 7 dpi A17 and DZA315). *Fom*_*15733* is predicted CDC encoded and shares greatest similarity with proteins from *F. oxysporum* f. sp. *raphani* (Brassica pathogen, 90 %) and f. sp. *pisi* (pea pathogen, 87 %) but low similarity with other *F. oxysporum* ff. spp. (e.g. 26−36 % with *ciceris*, *melonis, lycopersici*, *Fo*5176, see [5]). This protein contains a fungal specific transcription factor domain (Pfam:PF04082) and a fungal Zn(2)-Cys(6) binuclear cluster domain (Pfam:PF00172). The other transcription factors identified in the up-regulated datasets were only found in the DZA315 7 dpi dataset (*Fom*_*14279, 14162, 07202*), are predicted to reside on core chromosomes and share most similarity to proteins from other *F. oxysporum* ff. spp.. The *Fom* homologue (*Fom_08318*) of *Fol* transcription factor SIX Gene Expression 1 (*SGE1*) whose expression is up-regulated during infection of tomato roots and is required for expression of most secreted *Fol* effectors [41, 42], was detected as expressed in vitro but not detected as significantly up-regulated in our *in planta* DEG datasets.

**Fig. 4:**
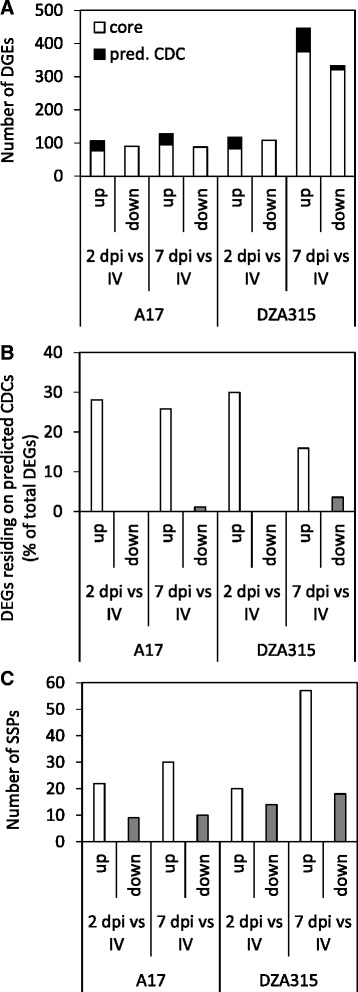
DEGs in up-regulated datasets contain an enrichment of genes located on putative non-core chromosomes and encoding predicted small secreted proteins. **a**-**b** Predicted putative chromosomal location of DEGs with **a**) total numbers in each dataset and **b**) percentage of DEGs predicted to reside on a predicted conditionally dispensable chromosome (CDC). **c** DEGs predicted to encode small secreted proteins within each dataset

Next we applied several criteria to identify candidate effector and host-specific pathogenicity genes. These included characteristics such as predicted secretion, *in planta* up-regulated gene expression and chromosomal location, as *F. oxysporum* effectors have previously been identified on non-core or conditionally dispensable chromosomes (CD chromosomes). The majority of DEGs in all datasets were located on scaffolds predicted to form part of core *Fom* chromosomes [5] (Fig. 4a). Most DEGs predicted to lie on putative CD chromosomes were only identified within the up-regulated datasets (Figs. 4a, b). DEGs encoding SSPs were also predominantly identified within the *in planta* up-regulated datasets (Fig. 4c, Additional files 2 and 3). Interestingly the number of SSPs in A17 datasets didn’t differ much between the two sampled time-points but in DZA315 the number almost tripled between the 2 and 7 dpi up-regulated datasets (Fig. 4c). Further, DEGs within the susceptible DZA315 datasets also contained a larger proportion of genes expressed more highly *in planta* and these were enriched for SSPs (Additional file 7).

Overall, the enrichment during the early stages of infection of genes encoding SSPs, uncharacterised proteins and proteins with roles in protein/sugar degradation and their transport, oxidative stress and other pathogenicity associated processes indicates substantial changes in pathogen gene expression upon colonisation of a susceptible host.

### Highly up-regulated genes unique to *in planta* up-regulated datasets from a susceptible or resistant host and links to pathogenicity

As we were most interested in identifying pathogenicity factors we focused firstly on the differences between genes expressed during infection of resistant or susceptible host accessions (indicating changes that *Fom* may undergo when it detects a susceptible or resistant host) and secondly on those that were expressed during both infection of a susceptible and a resistant host (indicating key roles in pathogen attack).

**Fig. 5:**
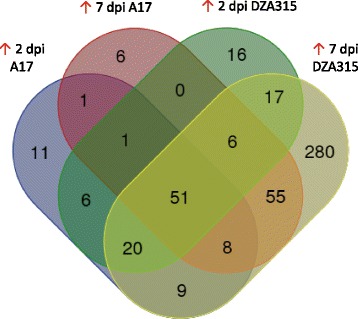
Unique and overlapping *Fom* DEGs between A17 and DZA315 *in planta* up-regulated datasets. Venn diagram of DEGs in overlapping A17 and DZA315 *in planta* up-regulated datasets. Red arrows indicate up-regulated *in planta*

Firstly we compared DEGs unique to infection of each host accession. Under this analysis, 11 and 6 DEGs were uniquely significantly up-regulated in A17 at 2 and 7 dpi respectively, while 16 and 280 were unique to DZA315 at the same time-points (Fig. 5). Of those expressed during infection of A17 at 2 dpi, 4 of the most highly induced (32-13,000-fold) were co-localised at the genomic scale (Table 3) and another, *Fom*_08477, is a predicted SSP showing similarity to an uncharacterized protein from the rice pathogen *Fusarium fujikori*. At 7 dpi in A17 all six unique DEGs were core scaffold located with two induced >100-fold and encoding SSPs (*Fom*_*05133*, *Fom*_*09362*, encoding a hypothetical protein and a glycoside hydrolase respectively).

**Table 3:** Examples of genomic co-localisation of *in planta* up-regulated differentially expressed *Fom* genes unique to DZA315 or A17 datasets. Full DEG lists are detailed in Additional files 2 and 3

Dataset	Gene IDs	Chrom./ scaffold^a^	Pfam domain / best BlastP match / other characteristics
A17 2 dpi	*Fom*_*11200, 11207, 11209, 11211*	core (scaffold_15; 1.38 Mb)	GST, MFS transporter, FAD-binding, uncharacterised protein
DZA315 7 dpi	*Fom_01388, 01394, 01397*	core (scaffold_2; 3.35 Mb)	glycoside hydrolase, fasciclin, MFS transporter
	*Fom_01935, 01936, 01938*	core (scaffold_2; 3.35 Mb)	arca-like protein, MFS transporter, glycoside hydrolase
	*Fom_03730, 03733, 03734*	core (scaffold_4; 2.62 Mb)	Hypothetical, MFS transporter, synthase
	*Fom_15853, 15855, 15856, 15860*	core (scaffold_131; 0.03 Mb)	FAD-binding protein, ADH, FAD-binding protein, p450
	*Fom_14230, 14231, 14232, 14241*	CDC (scaffold_31; 0.21 Mb)	Oxidoreductase, MFS transporter, FAD-binding protein, oxidase

^a^predicted core or CD chromosome (CDC) location and scaffold size based on [5]

The 16 unique DEGs from the DZA315 2 dpi dataset comprised of both core and non-core located genes with inductions ranging from 3.5 to >65,000-fold over in vitro conditions and encoding several FAD-binding proteins, hydrolases, peptidase, MFS transporter and p450 amongst others. Of the 280 significant DEGs in the DZA315 7 dpi unique dataset the majority were core scaffold located (243) and 43 were up-regulated *in planta* over 100-fold. Several were also co-localised (Table 3). Two *Fom* SSPs (*Fom*_08816, *Fom*_11981) with similarity to the phytotoxin cerato-platanin were also only identified in the DZA315 *in planta* 7 dpi up-regulated dataset. Some cerato-platanin members in other phytopathogens are implicated in disruption of host cell walls through expansin-like activity, chitin oligomer sequestration and the ability to induce plant cell necrosis [43, 44]. Other proteins only upregulated in DZA315 at 7 dpi included a xylosidase arabinosidase (*Fom*_*12400*) and an l-arabinitol 4-dehydrogenase (*Fom_00399*) with implied roles respectively in the hydrolysis of major hemicellulose component xylan to xylose and other sugars, and the catabolism of L-arabinose, an important constituent of plant cell wall polysaccharides.

### Overlapping highly *in planta* up-regulated genes in susceptible and resistant host accessions and links to pathogenicity

Analysis of DEGs common to all four *in planta* up-regulated datasets identified 51 significantly induced DEGs (Fig. 5 and Table 4). These included 17 SSPs, 11 of which were predicted as effectors by EffectorP (8 CD and 3 core scaffold encoded). Thirty genes were predicted to be encoded on core scaffolds with 1/3 of these (21) not predicted as effectors by EffectorP and predominantly composed of proteins functioning in protein degradation and the breakdown of plant cell walls (hydrolases, peptidase, pectinesterase), transport or electron transfer (MFS transporter, p450s) or oxidative processes (oxidoreductases).

**Table 4:** Differentially expressed *Fom* genes significantly up-regulated *in planta* during infection of DZA315 or A17 at 2 and 7 dpi. DEGs are ranked by DZA315 fold change (FC) over in vitro at 2 dpi. Bold FC indicates DZA315 fold changes greater than A17

Protein ID	Chrom./scaffold^a^	Sec. pred.^b^	SSP^c^	Eff.P pred.^d^	AA^e^	Potential role^f^	DZA315 FC^g^		A17 FC^g^		IV ^h^
							2dpi	7dpi	2dpi	7dpi	
Fom_SIX13	CDC	+	SSP	-	**292**	SIX effector	**397336**	**1290948**	280959	1123836	-
Fom_16257	CDC	+	SSP	+	**91**	uncharacterised	**172951**	**741455**	150562	301124	-
Fom_16301	CDC	+	SSP	+	**144**	LysM	**161369**	**912838**	114105	425854	-
Fom_16235	CDC	+	SSP	+	**264**	uncharacterised	**37641**	**172951**	35120	131072	+
Fom_15517	CDC	-			567	abhydrolase/peptidase	**12417**	7132	7643	8780	+
Fom_16729	core	-			**54**	Protease (partial)	11585	**212927**	23170	114105	-
Fom_16326	CDC	+	SSP	+	**111**	uncharacterised	**11585**	**106464**	7132	57052	+
Fom_SIX1	CDC	+	SSP	-	**279**	SIX effector	**11585**	**61147**	10809	40342	+
Fom_SIX8	CDC	+	SSP	+	**141**	SIX effector	**8780**	**106464**	8192	70240	+
Fom_16154	CDC	+		-	610	GMC_oxidoreductase	**4390**	**20171**	3566	12417	+
Fom_16208	core	-			**185**	peptidase	**2896**	**37641**	2048	15287	+
Fom_12303	core	+	SSP	+	**169**	uncharacterised	2195	**61147**	2353	23170	-
Fom_16263	CDC	-			**235**	uncharacterised	**2195**	**14263**	1911	6208	+
Fom_15948	CDC	+	SSP	+	**215**	peroxidase	**1911**	**16384**	1097	6654	+
Fom_10610	core	+		-	478	oxidoreductase	**1552**	**5793**	1448	2896	+
Fom_16592	CDC	-			**87**	uncharacterised	**1261**	**5043**	630	3566	+
Fom_15730	CDC	-			**199**	GST	**776**	**891**	315	588	+
Fom_15949	CDC	-			373	peroxidase	776	**8192**	776	4096	+
Fom_09820	core	-			545	p450	**512**	239	388	239	+
Fom_09550	core	-			**87**	uncharacterised	446	3566	1097	3822	+
Fom_SIX9	CDC	+	SSP	+	**122**	SIX effector	**239**	**1552**	208	1024	+
Fom_15614	CDC	+	SSP	-	**221**	glyco_hydrolase	**223**	**478**	181	274	+
Fom_06101	core	-			420	uncharacterised	**194**	**239**	181	128	+
Fom_14503	core	-			**138**	SnoaL	169	**119**	223	45	+
Fom_07856	core	-			533	MFS	**147**	**169**	97	84	+
Fom_16718	core	-			**79**	protease	111	**2702**	208	1176	+
Fom_07866	core	-			**123**	uncharacterised	**79**	**69**	60	34	+
Fom_16085	CDC	-			403	2OG-FeII_Oxy	**60**	**446**	52	315	+
Fom_10284	core	-			**112**	ligase	**60**	239	49	338	+
Fom_02574	core	+	SSP	+	**87**	uncharacterised	**49**	**32**	32	24	+
Fom_04867	core	-			**300**	NAD_binding	**39**	**23**	32	9	+
Fom_07859	core	-			**292**	LigB, dioxyenase	**37**	**17**	11	11	+
Fom_01704	core	-			398	methionine synth.	24	**26**	32	23	+
Fom_13701	core	-			391	oxidoreductase	**24**	**24**	20	11	+
Fom_12076	core	+		-	324	uncharacterised	21	**45**	26	20	+
Fom_02353	core	+	SSP	-	**295**	endoglucanase	21	**39**	23	23	+
Fom_15788	CDC	+	SSP	+	**199**	uncharacterised	**18**	**69**	11	49	+
Fom_04087	core	+	SSP	+	**158**	uncharacterised	**16**	**91**	13	42	+
Fom_10931	core	+		-	330	pectinesterase	11	**60**	20	26	+
Fom_08395	core	+	SSP	-	**291**	abhydrolase	**9**	**17**	6	5	+
Fom_16385	CDC	-			**195**	glyco_hydrolase	9	**39**	10	30	+
Fom_07662	core	-			438	aminotransferase	**9**	**34**	4	16	+
Fom_08559	core	+		-	433	glyco_hydrolase	8	**64**	9	37	+
Fom_04883	core	-			514	p450	7	**9**	7	5	+
Fom_15521	CDC	-			660	AMP-binding	**6**	5	5	5	+
Fom_05481	core	+	SSP	-	292	uncharacterised	**6**	**5**	5	3	+
Fom_01040	core	-			124	uncharacterised	6	7	12	8	+
Fom_16221	CDC	-			83	glyco_hydrolase	5	**37**	6	23	+
Fom_16350	core	-			174	abhydrolase	5	2	5	4	+
Fom_10260	core	-			255	flavodoxin	**5**	**10**	3	5	+
Fom_09704	core	+		-	418	glyco_hydrolase	4	**32**	4	17	+

^a^predicted core or CD chromosome (CDC) location

^b^predicted secreted based on SignalP

^c^predicted small secreted protein (SSP) based on ≤ 300 amino acids, SignalP, and ≤1 transmembrane domains

^d^EffectorP prediction (performed if predicted to be secreted): + effector, - non-effector

^e^Bold font: protein length ≤ 300 amino acids

^f^Potential role based on Pfam domain, best BlastP match or other characteristics

^g^Fold change *in planta* gene expression over in vitro conditions

^h^expression in vitro, –: 0-10 % RNA-Seq read coverage over all replicates, +: >10 % RNA-Seq read coverage over all replicates

**Fig. 6:**
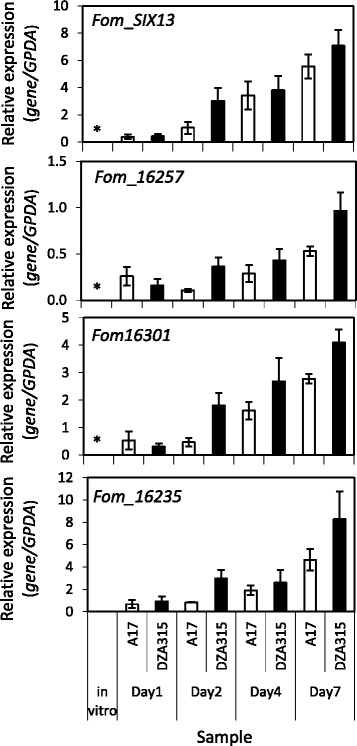
qRT-PCR validation of candidate effector genes in vitro versus *in planta* over an infection time-course. Expression of candidate effector genes as determined by qRT-PCR in in vitro samples and *M. truncatula* DZA315 and A17 root samples harvested at 1, 2, 4 and 7 days post inoculation (dpi) with *Fom*. in vitro samples are averages ± SE of 3 biological replicates. *In planta* samples are averages ± SE of 4 biological replicates each consisting of pools of 10 seedlings. Gene expression levels are relative to the fungal *GPDA* gene (*Fom*_*05751*). Note: * No detectable expression in any in vitro replicates

We first assessed the overlapping DEGs for the presence of those encoding the well documented *F. oxysporum Secreted In Xylem* (*SIX*) proteins, with known roles in virulence and/or avirulence described for some in *F. oxysporum* f. sp. *lycopersici, F. oxysporum* f. sp. *melonis* or *Fo*5176 (Brassica infecting) [6, 13, 14, 17, 18, 45–49]. SIX proteins can broadly be defined as small, generally cysteine rich, and possessing a secretion signal [13, 49]. Of the 14 *F. oxysporum* f. sp. *lycopersici* SIX proteins described so far, all four that we previously identified in the *Fom* genome (*Fom SIX1, SIX8, SIX9* and *SIX13*) [5] were significantly upregulated in all *in planta* datasets where they were amongst the top *in planta* induced DEGs. *Fom*_*SIX13* was the most highly induced at levels 1 million times greater than in vitro (Table 4). qRT-PCR verification of *Fom SIX* gene expression over a 1 to 7 day infection time-course revealed all but *Fom*_*SIX13* could be reliably detected under in vitro conditions suggesting expression of this *SIX* gene in *Fom* responds specifically to detection of a possible host (Fig. 6, Additional file 8). Correlating with our RNA-Seq data (Table 4), qRT-PCR examination of all four *Fom SIX* genes confirmed these genes were overall more highly expressed in DZA315 than A17. Interestingly *Fom*_SIX13 and *Fom*_SIX1 didn’t meet the statistical cut-off of the EffectorP [38] prediction algorithm (as also observed for putative *Fol* effectors SIX7 and SIX13).

After *Fom*_*SIX13*, the next three most highly *in-planta* induced DEGs are strong effector candidates. These encode *Fom*_*16257* a *Fom*-unique protein with unknown function, *Fom*_*16301* a SSP encoding a LysM-domain (~40 residue lysin motif implicated in chitin binding [50–52]), and *Fom*_*16235* an uncharacterised protein with orthologs in other *F. oxysporum* ff. spp.. qRT-PCR verification of *Fom_16257* expression confirmed increased expression in DZA315 over A17 and absence of expression under in vitro conditions (Fig. 6). Similar results were observed for *Fom*_*16301*. Interestingly, *Fom*_*16235* was lowly expressed under in vitro conditions. Along with *Fom*_*16301*, another LysM-domain containing SSP gene (*Fom*_*04092*) was also significantly upregulated, but only at 7 dpi. The *Fom*_*16301* encoded protein contains one LysM domain, sharing best similarity to a protein from *F. oxysporum* f. sp. *pisi* (95 %), other *F. oxysporum* ff. spp. and *Colletotrichum* species. *Fom*_04092 contains two LysM domains and is most similar to proteins from other *F. oxysporum* ff.spp. and the rice pathogen *F. fujikuroi*.

Other highly *in planta* up-regulated genes included three proteases/peptidases and genes involved in oxidative stress responses (Table 4). Of the latter type, *Fom*_*15948* and *Fom*_*15949* both encode peroxidases predicted to reside alongside each other. *Fom*_15948 is a predicted effector and SSP sharing 95 % identity to other *F. oxysporum* peroxidases/catalases or hypothetical proteins, as well as to proteins from *F. solani*, *F. fujikori*, and *Colletotrichum* and *Verticillium* species.

Several uncharacterised proteins with properties of effectors were also identified as highly up-regulated *in-planta* during infection of both resistant and susceptible *M. truncatula* accessions (Table 4). Of those predicted as SSPs, only two were not predicted as effectors by EffectorP and these encode a putative endoglucanase (*Fom*_02353) and a cupredoxin domain-containing protein (*Fom*_05481) sharing 94-96 % similarity with several other serine rich proteins from *F. oxysporum* ff. spp. The serine rich nature of this protein may imply possible involvement in post-translational regulatory processes or protein-protein interactions. The remaining novel uncharacterised effector-like proteins identified contain no other known functional protein domains.

## Discussion

The success of a pathogen’s attack requires the co-ordinated early expression of its pathogenicity arsenal. While an increasing list of putative arsenal components from various *F. oxysporum* ff. spp. have been predicted via whole genome sequencing or more classical PCR-based approaches, the relatively low abundance of fungal transcripts in host root tissues during early stages of infection has meant that very few whole transcriptome based identification studies have been accomplished. To overcome the constraint of low *F. oxysporum* RNA abundance in *M. truncatula* root tissues early in infection, we coupled our draft *Fom* genome assembly [5] and the pathogen’s vegetative (in vitro) transcriptome with high coverage *in planta* RNA-Seq of inoculated host roots to identify genes specifically up-regulated upon host infection. Comparison of a susceptible versus a resistant *M. truncatula* accession at 48 h post infection and at the later time-point of 7 dpi when disease symptoms on susceptible *M. truncatula* plants start to manifest, facilitated the identification of DEGs either unique or common to resistant or susceptible *F. oxysporum*-legume host interactions. These studies revealed a greater degree of transcriptional reprogramming during the susceptible plant interaction including expression of genes encoding SSPs and genes associated with plant cell wall and membrane degradation, nutrient uptake and oxidative processes. In addition we identified novel candidate effectors and pathogenicity-associated genes highly induced during infection of both susceptible and resistant plants.

To date, several transcriptome studies (e.g. RNA-Seq, ESTs/cDNAs) examining the *F. oxysporum*-host interaction have been reported, including those from infected roots of *Arabidopsis*, cabbage, banana, melon, cotton, chickpea, soybean or *Medicago*. The majority of these studies only included comparative analyses amongst plant transcripts due to insufficient sequencing depth for fungal transcript detection [19, 31, 48, 50–59]. Transcriptome analysis of this pathogen is also hindered by inherent variations in *F. oxysporum* root-colonisation dynamics at early stages of host colonisation and subsequent variations in disease progression amongst individual plants. Those studies with probes unique to the pathogen or enough coverage to detect pathogen transcripts are limited and have typically involved analysis of later time-points in the pathogen-host interaction process. For example, Schmidt and colleagues [48] analysed the expression of *F. oxysporum* f. sp. *melonis* transcripts at 10 dpi on melon roots. Here we were able to overcome these constraints by pooling root tissues from *Fom* inoculated *M. truncatula* seedlings and combining high coverage RNA-Seq with our stringent DEG selection criteria.

In addition to *in planta F. oxysporum* transcriptome analysis, *in planta* proteomics has been used to identify *F. oxysporum* pathogenicity components. The combined studies of Rep, Houterman, Schmidt and their colleagues [13, 49] which identified several avirulence and virulence products of *Fol* in the xylem sap proteome of infected tomato plants, were the first to identify the key Secreted in Xylem (SIX) effectors. Two studies have been conducted on the root proteomes of *F. oxysporum* infected legumes (chickpea-*F. oxysporum* f. sp. *ciceris* [24] and pea-*F. oxysporum* f. sp. *pisi* [60]) however, neither of these studies assessed the presence of *Fusarium* proteins.

Via RNA-Seq Guo and colleagues [19] were successful in comparing the vegetative transcriptomes of two races (virulent and avirulent) of *F. oxysporum* f. sp. *cubense* against their transcriptomes at 2 days post-infection of Cavendish banana. As with our *in planta* up-regulated *Fom* analysis, Guo and colleagues [19] found the largest groups of expressed genes were significantly enriched for catalytic and metabolic activities. However, the next most enriched GO term assignments differed between our studies, with the *F. oxysporum* f. sp. *cubense* datasets enriched for primary/cellular/nitrogen compound metabolic and biosynthetic processes, while *Fom in planta* datasets were most enriched for binding/catabolism/biosynthesis/and hydrolase activity, followed by cell/carbohydrate metabolism/and nucleic acid metabolism (Additional file 4). This suggests that at the early time-point of 2 dpi of *Medicago* roots there is a substantial bias towards host cell wall and membrane degradation and the metabolism of released nutrient sources such as sugars. Furthermore, these processes were more pronounced during the susceptible *M. truncatula* DZA315-*Fom* interaction (Fig. 3) even though at 2 dpi the degree of *Fom* colonisation was not significantly different between the susceptible DZA315 and resistant A17 *M. truncatula* hosts (Fig. 1e). It is interesting to speculate that differences observed between *Fom* and *F. oxysporum* f. sp. *cubense* may potentially be due to differences in root tissue or cell structure between a dicot/legume or monocot host.

Functional analysis revealed enrichment during infection of susceptible DZA315 plants of *Fom* DEGs encoding protein domains (Pfam) associated with degradation of proteins and carbohydrates/sugars as well as membrane transport and oxidative processes (Fig. 3). Lower numbers of these protein features were detected in the A17 up-regulated DEG datasets, with more pronounced differences observed at 7 dpi when a significant increase in *Fom* biomass was recorded in susceptible plants (Fig. 1e). Like other pathogenic plant-fungal pathogens, *F. oxysporum* genomes are enriched in plant cell wall degrading enzymes (CWDEs) [5, 9, 61–65] and are known to secrete these extracellular degradative enzymes during host colonization [66, 67]. These include enzymes such as polygalacturonases, pectate lyases, xylanases and proteases to break down cell walls and membranes to release nutrient sources such as sugars [20, 68] with expression during infection and roles in virulence demonstrated for some via double deletion mutants (e.g. double *Fol polygalacturonase* and *endopolygalacturonase Δpg1Δpgx6* deletions) [19, 31, 64, 69, 70]. *Fom* genes highly expressed during infection of susceptible plants included several proteases/peptidases (including metallo- and serine-proteases) and pectate lyases with proposed roles in degradation of pectin and other cell wall components. Recently it was demonstrated that a secreted *Fol* metalloprotease and serine protease could cleave tomato chitinases with double deletion mutants of these two genes impaired in virulence on tomato [71]. Other *Fom* highly *in planta* expressed genes included several glycoside hydrolases that may act on the substrates sucrose, pectin, hemicellulose and chitin (Fig. 3). Fungal chitinases belong exclusively to the glycosyl hydrolase (GH) 18 family, but apart from a fundamental role in chitin hydrolysis little is known about their role during pathogen-plant interactions [72, 73]. It is suggested that during host infection they may have roles in fungal cell wall remodelling including involvement in processes such as spore germination, hyphal tip growth and branching [72, 73]. Transcripts encoding proteins with roles in membrane transport such as permeases, and sugar, amino acid and Major Facilitator Superfamily (MFS) transporters were also highly up-regulated and enriched in the DZA315 DEG datasets implying enhanced uptake of nutrient sources such as sugars released from plant roots by the aforementioned enzymes.

Cytochrome p450s were overrepresented in the *in planta* up-regulated *Fom* DEG datasets suggesting important roles in pathogenicity, with two identified in the overlapping datasets (Table 4). Located adjacent to one of these (*Fom_09820*) is a putative effector (*Fom_09819*) whose expression was up-regulated in both A17 and DZA315 7 dpi and shares 69 % protein identity to the *Verticillium dahliae* isochorismatase effector VdIsc1 which is required for full pathogenesis and disrupts plant defense responses by interfering with salicylate production [74]. Three oxidoreductases and two peroxidases (including EffectorP predicted *Fom*_*15948*) were identified in the overlapping *in planta* up-regulated DEG dataset (Table 4). A secreted *Fol* catalase-peroxidase (FOXG_17460) and an oxidoreductase (Orx1) were detected in the xylem sap proteome of *Fol*-infected tomato plants suggesting they are important for infection [13, 49]. *Fom*_15948 shares 84 % amino acid identity with FOXG_17460 and similar levels of identify with other *F. oxysporum* ff. spp. catalase/peroxidases, but differs by only one amino acid from a predicted catalase/peroxidase from the pea-infecting *F. oxysporum* f. sp. *pisi* HDV247 (FOVG_19731). Other genes expressed within the DZA315 7 dpi unique dataset included hydrolases, oxidases, oxidoreductases, phytotoxic cerato-platanins and several cytochrome p450s with similarity to pisatin demethylases (PDAs) (*Fom*_*15860*, *Fom*_*09645*, *Fom*_*13458*) that may degrade phytoalexin pterocarpans produced by *Medicago* species. While some of these genes were detected during infection of A17 they did not meet the minimum read coverage criteria.

A significant proportion of the *in planta* up-regulated *Fom* DEGs were enriched for proteins with no functional annotation including the tripling of abundance of uncharacterised genes in DZA315 infection between 2 and 7 dpi. We applied several criteria to narrow in on proteins within these datasets that may have roles in host-specific pathogenicity. One of these, up-regulated *in planta* expression during host infection, is a stringent criteria that has been successfully applied to identify and/or verify avirulence (Avr) proteins and putative effectors in other ff. spp. of *F. oxysporum* [5, 6, 13, 19, 32, 33, 65, 75]. For example, expression of *Fol* Avr3 (also referred to as SIX1) and *Fol* SIX6 whose gene products are respectively recognised by the tomato *F. oxysporum Resistance* gene *I3* or suppress *I2*-mediated cell death, are strongly induced in the presence of living plant cells [17, 32, 41]. It should be noted that not all fungal effectors are exclusively expressed *in planta* (reviewed in [33, 65]) and we therefore did not ignore any potential effector candidates that were expressed in our in vitro dataset. We also placed emphasis on genes likely to be SSPs and CDC encoded as in the genus *Fusarium* CDCs are often enriched in rapidly evolving genes, can be horizontally transferred and encode novel effector candidates detected in the xylem sap of *Fol* infected tomato plants [9, 10, 13, 76]. The majority of candidate effectors we identified from our overlapping expression analysis (Table 4) were either unique to *Fom* and thus may define its host specificity, or were conserved to some degree with proteins of other *F. oxysporum* ff. spp. or other *Fusaria* and thus may have conserved roles in plant pathogenicity. We identified two proteins (*Fom*_16301, *Fom*_15948) that share a high degree of similarity to proteins from anthracnose disease causing *Colletotrichum* species. This similarity has been reported previously between effectors from *Fol* or *F. oxysporum* f. sp. *melonis* (e.g. SIX6) or candidate effectors from other *Fusaria* [17, 76]. *Fom*_16301 contains a lysin motif (LysM) and shares some similarity to extracellular protein7 (Ecp7) of unknown function from the tomato fungal pathogen *Cladosporium fulvum* and a LysM containing effector candidate from *F. oxysporum* f. sp. *melonis* [48, 77]. It is suggested that other *C. fulvum* effectors containing the LysM-domain may suppress chitin triggered immunity by protecting fungal hyphae against host chitinases or sequestering fungal cell wall derived chitin fragments from host detection [77–80]. The *Fom* genome encodes 11 proteins with LysM-domains and in addition to up-regulated *in planta* expression of the predicted CDC encoded *Fom*_*16301*, we also identified a core chromosome encoded LysM-domain containing protein (*Fom*_04092) sharing this expression profile. The vascular wilt pathogen *Verticillium dahliae* also contains an expanded LysM effector family but the expression of only one of these is induced *in planta* [81]. This effector, VDAG_05180, is CDC encoded and contributes to virulence on tomato, while the remaining LysM-domain proteins are core chromosome located, not expressed during infection and single deletion mutants have no effect on virulence [18, 81]. While the roles of the latter putative *V. dahliae* LysM effectors remains ambiguous, the *in planta* up-regulated expression of *Fom*_*16301* and *Fom*_*04092* suggests these two SSPs may contribute to pathogenicity.

The most highly *in planta* up-regulated gene detected in all *Fom* datasets (Table 4) was *Fom*_*SIX13* whose expression could not be detected under vegetative conditions by either RNA-Seq or qRT-PCR (Fig. 6). The functional role of SIX13 homologues across *F. oxysporum* ff. spp. is unknown. Previously we found *Fom*_SIX13 shares most similarity with a SIX13 homologue from a melon-infecting *F. oxysporum* isolate and less similarity with its legume-infecting counterparts in f. sp. *pisi* or f. sp. *ciceris*, although it was the only *SIX* gene common to the legume-infecting ff. spp. analysed [5]. Recently, additional SIX13 sequences have been identified in other *F. oxysporum* f. sp. *pisi* isolates (races 2 and 5) [75] and BLASTN analysis [82] revealed the *Fom*_*SIX13* gene sequence shares 98 % identity with these two f. sp. *pisi SIX13* sequences suggesting potentially conserved roles on legume hosts. Within the overlapping *in planta* up-regulated DEGs we also identified seven genes unique to *Fom* and sharing no orthologues with other fungal species. This includes three uncharacterised proteins, a glycoside hydrolase, two proteases and a peptidase (Table 4 and Additional files 2 and 3). Of these, the CDC encoded SSP *Fom*_16257 contains a motif resembling a zinc finger domain suggesting this protein may bind host DNA sequences [5]. These features combined with its expression only during infection, earmarks *Fom*_*16257* as a host-specific effector for future functional studies and identification of its possible host target(s).

## Conclusions

In summary, our findings greatly improve the current understanding of *Fusarium* wilt pathogen molecular responses during early stages of legume colonisation and infection. Our analysis of *in planta* up-regulated genes represents a significant resource that can be leveraged to hone in on candidate effectors and pathogenicity associated genes from other *F. oxysporum* ff. spp.. Prioritisation for functional follow up studies to elucidate the role(s) of candidate *Fom* effectors listed within Table 4 will focus on those that are *Fom* specific and thus have implied roles in host specificity or those that are highly conserved amongst other legume infecting *F. oxysporum* ff. spp. and will expedite the dissection of their pathogenicity mechanisms on important global legume crops.

## Methods

### Isolate sources and growth conditions

*F. oxysporum* f. sp. *medicaginis* (Weimer) W.C. Snyder & H.N. Hansen, (*Fom*-5190a, BRIP 5190a/IMI 172838, collection number 19911) was obtained from the Queensland Plant Pathology Herbarium (BRIP) and has been described previously [5]. *Fom* was maintained on sterile filter paper and grown on ½ strength potato dextrose agar at 22 °C in the dark. *M. truncatula* accessions A17 and DZA315 were at least second generation inbred lines derived from seed obtained from the South Australian Research and Development Institute (SARDI). *M. truncatula* seeds were germinated on damp filter paper, transplanted into 30 mm Jiffy-7 peat pellets and grown under a short day-light regime (8-h light/16-h dark) at 21 °C.

### *F. oxysporum* disease assays

*Fom* disease assays of *M. truncatula* were conducted as described previously [5, 29]. Briefly, a 1 × 10^6^ spores mL^–1^ spore suspension was used to inoculate the roots of two week old seedlings which had roots protruding from the peat pellets trimmed prior to inoculation. Seedlings in peat pellets were placed in a petri dish of spore suspension for 5 min, followed by addition of a further 1 mL of spore suspension to the base of the hypocotyl. Inoculated pellets were transferred to growth trays lined with a plastic sheet and a thin layer of damp vermiculite, covered with a clear plastic dome to maintain humidity, and incubated under a long-daylight regime (16-h light/8-h dark) at 28 °C. Plants were scored every 7 days over 4 weeks to assess disease progression.

### RNA isolation

RNA for qRT-PCR and RNA-Seq experiments on *Fom* infected *M. truncatula* root tissue was conducted as described previously [5]. Briefly, root tissue was collected from 10 plants and pooled per replicate at 1, 2, 4 and 7 days post inoculation. For *Fom* in vitro samples, mycelia were grown in a petri dish containing one-half-strength potato dextrose broth for 7 days at 22 °C and mycelia harvested by filtering through Miracloth. RNA extraction was performed independently for each of 3 replicates using a Trizol extraction (Sigma-Aldrich, St. Louis, MO) followed by DNase treatment using TURBO DNase (Ambion). RNA samples were cleaned via RNeasy mini spin columns (Qiagen).

### qRT-PCR

Following RNA isolation and DNase treatment, complementary DNA synthesis was performed with 1ug of input RNA followed by qRT-PCR performed using SsoFast EvaGreen Supermix (Bio-Rad) on a CFX384 (Bio-Rad) system as described previously [5]. Absolute gene expression levels relative to *F. oxysporum* housekeeping gene *GPDA* (*Fom_05751*) were used for each complementary DNA sample using the equation: relative ratio gene of interest/GPDA = (Egene^-Ct gene^)/(EGPDA^-Ct GPDA^) where Ct is the cycle threshold value. *Medicago* root samples were verified for even abundance of plant input material using the *M. truncatula B-tubulin* reference gene [5, 83] which was found to be within ±1 Ct across all samples. Quantification of *Fom* biomass in *M. truncatula* tissues was conducted as described previously by determining the abundance of *Fom*_*18S* relative to *M. truncatula*_*18S* [5]. Primer sequences have been previously published [5] and/or are also listed in Additional file 9.

### RNA-Seq library construction, Illumina sequencing and read-mapping

RNA integrity was confirmed using the Agilent 2100 Bioanalyser Plant Nano system (Agilent Biotechnologies). Stranded Illumina TruSeq libraries were generated from 1 μg of total RNA and sequenced (100 bp paired end reads) on an Illumina HiSeq platform by the Australian Genome Research Facility (AGRF). As per [5], RNA-Seq paired-end reads were trimmed for low-quality base-calls (≥ q30) and Illumina adapter sequences using Cutadapt v1.1 [84] (parameters: --quality-cutoff 30 --overlap 10 --times 3 –minimum-length 25) with reads trimmed to less than 25 bp discarded and remaining reads sorted into pairs and singleton reads. RNA-Seq reads were then mapped to the *Fom*-5190a genome assembly via Tophat2 (TopHat2 v2.0.9) (parameters: --b2-very-sensitive -r 80 --mate-std-dev 40 -i 20 -I 4000 -g 20 --report-secondary-alignments --report-discordant-pair-alignments -m 0 --min-coverage-intron 20 --microexon-search --library-type fr-firststrand) [30]. BAM files generated from Tophat2 output were sorted using SAMtools version 0.1.19 [85] and visualised using IGV (version 2.3) [86]. Trimmed sequencing data is available from the NCBI/GenBank database under BioProject number PRJNA294248 (http://www.ncbi.nlm.nih.gov/bioproject/?term=PRJNA294248).

### Differential gene expression analysis

BAM files generated by TopHat2 [30] were used by BioKanga maploci [http://sourceforge.net/projects/biokanga/files/] to assign aligned reads to known loci. This step was followed by the genDEseq subprocess of BioKanga to generate counts files for differential expression. Read counts from different biological replicates and samples were combined for each gene and the resulting count matrix was normalized and analysed for differential expression using the EdgeR [34] package in R version 3.2.2 http://www.r-project.org/ using a false discovery rate cutoff of 0.05 and a minimum fold change of log2 ≥ 1. Identification of unique or overlapping genes within the DEG datasets and the generation of Venn diagrams was determined using Draw Venn Diagram http://bioinformatics.psb.ugent.be/webtools/Venn/ (accessed 12-01-16). Gene coverage was calculated from TopHat2 mapped RNA-Seq reads using BEDTools (v2.21.0) coverageBed [87].

### Protein characteristics

Protein characteristics were previously reported by [5] with GO term counts determined via CateGOrizer [88] (accessed 15-01-16). Statistical examination of over-represented protein functional attributes based on the number of proteins with specific Pfam domains were compared between DEG datasets and those from the whole annotated *Fom* genome using Fisher’s exact test with a significance threshold of *p* ≤ 0.05.

## Additional files

Additional file 1: Confirmation of disease progression in experiment from which plants were sampled for RNA-Seq. (PPTX 42 kb)
Additional file 2:
*Fom* genes up or down-regulated in DZA315 plants versus in vitro. (XLSX 464 kb)
Additional file 3:
*Fom* genes up or down-regulated in A17 plants versus in vitro. (XLSX 167 kb)
Additional file 4: Proportion of *Fom* proteins in A17 and DZA315 DEGs *in planta* up-regulated datasets with Gene Ontology (GO) assignments. (PPTX 165 kb)
Additional file 5: Gene Ontology (GO) assignments associated with *in planta* upregulated DEGs. (XLSX 13 kb)
Additional file 6: Pfam domains more abundant in the *in planta* up-regulated DEG datasets. (XLSX 17 kb)
Additional file 7: Plots of DEG significance versus *in planta* up-regulated fold induction. (PPTX 77 kb)
Additional file 8: qRT-PCR validation of candidate pathogenicity-associated genes in vitro versus *in planta* over an infection time-course. (PPTX 556 kb)
Additional file 9: qRTPCR_primers. (XLSX 9 kb)

## Abbreviations

CDC(s): Conditionally dispensable chromosome(s)
DEG(s): Differentially expressed gene(s)
dpi: Days post inoculation
f. sp: forma specialis
ff. spp: formae speciales
*Fol*: *F. oxysporum* f. sp. *lycopersici*
*Fom*: *F. oxysporum* f. sp. *medicaginis*
RNA-Seq: RNA sequencing
SIX: Secreted in xylem
