# Supplementary material for: Transcriptome analysis of the fungal pathogen Fusarium oxysporum f. sp. medicaginis during colonisation of resistant and susceptible Medicago truncatula hosts identifies differential pathogenicity profiles and novel candidate effectors
Source: BMC Genomics. 2016 Nov 3;17:860. doi: 10.1186/s12864-016-3192-2 (PMC5094085; doi:10.1186/s12864-016-3192-2)
Supplement: Additional file 1: — Confirmation of disease progression in experiment from which plants were sampled for RNA-Seq. (PPTX 42 kb) [file 12864_2016_3192_MOESM1_ESM.pptx]

## Slide 1
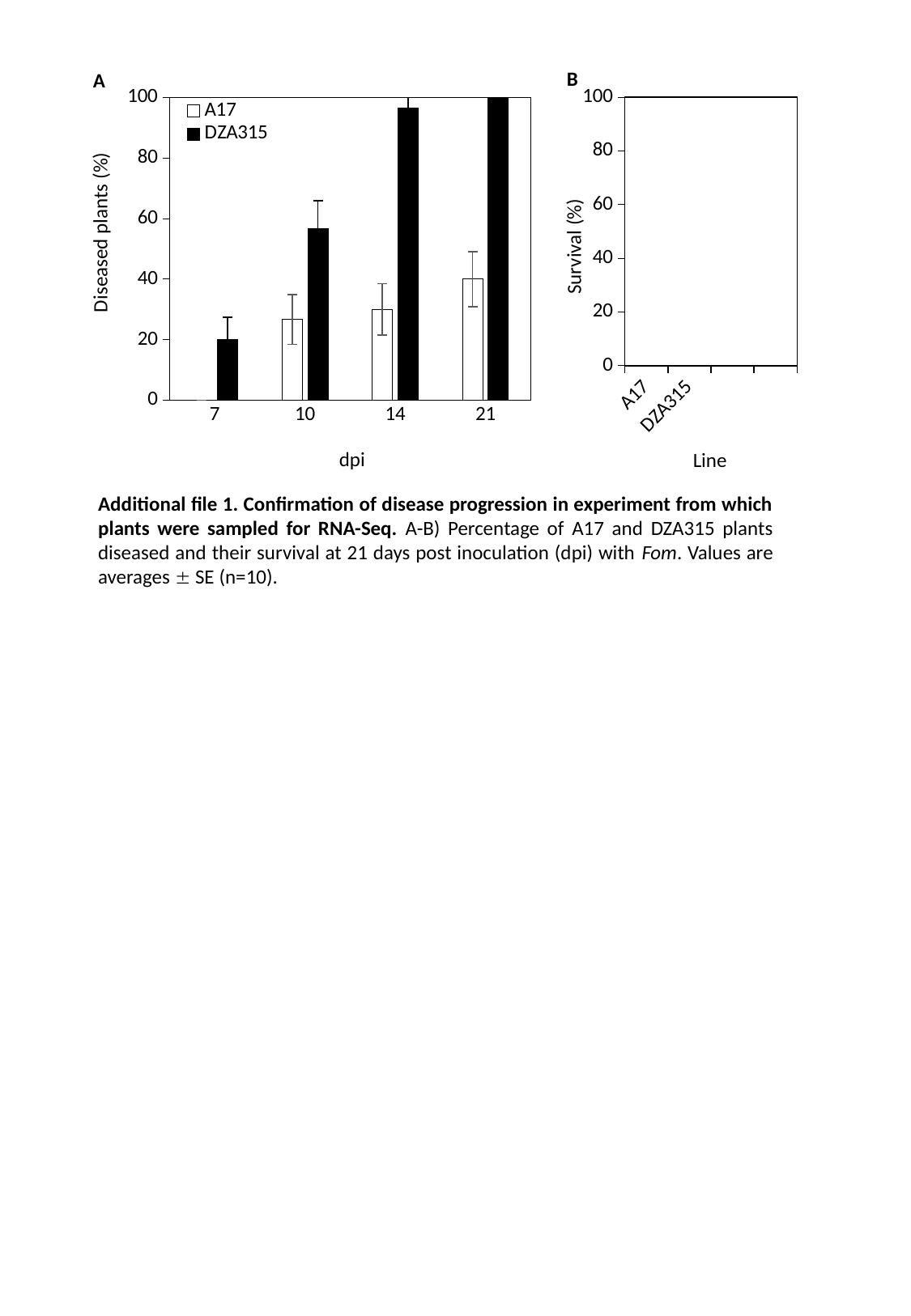

B
A
### Chart
| Category | A17 | DZA315 |
|---|---|---|
| 7 | 0.0 | 20.0 |
| 10 | 26.666666666666668 | 56.666666666666664 |
| 14 | 30.0 | 96.66666666666667 |
| 21 | 40.0 | 100.0 |
### Chart
| Category | avg |
|---|---|
| A17 | 100.0 |
| DZA315 | 23.333333333333332 |Diseased plants (%)
Survival (%)
dpi
Line
Additional file 1. Confirmation of disease progression in experiment from which plants were sampled for RNA-Seq. A-B) Percentage of A17 and DZA315 plants diseased and their survival at 21 days post inoculation (dpi) with Fom. Values are averages ± SE (n=10).
