# Supplementary material for: Transcriptome analysis of the fungal pathogen Fusarium oxysporum f. sp. medicaginis during colonisation of resistant and susceptible Medicago truncatula hosts identifies differential pathogenicity profiles and novel candidate effectors
Source: BMC Genomics. 2016 Nov 3;17:860. doi: 10.1186/s12864-016-3192-2 (PMC5094085; doi:10.1186/s12864-016-3192-2)
Supplement: Additional file 4: — Proportion of Fom proteins in A17 and DZA315 DEGs in planta up-regulated datasets with Gene Ontology (GO) assignments. (PPTX 165 kb) [file 12864_2016_3192_MOESM4_ESM.pptx]

## Slide 1
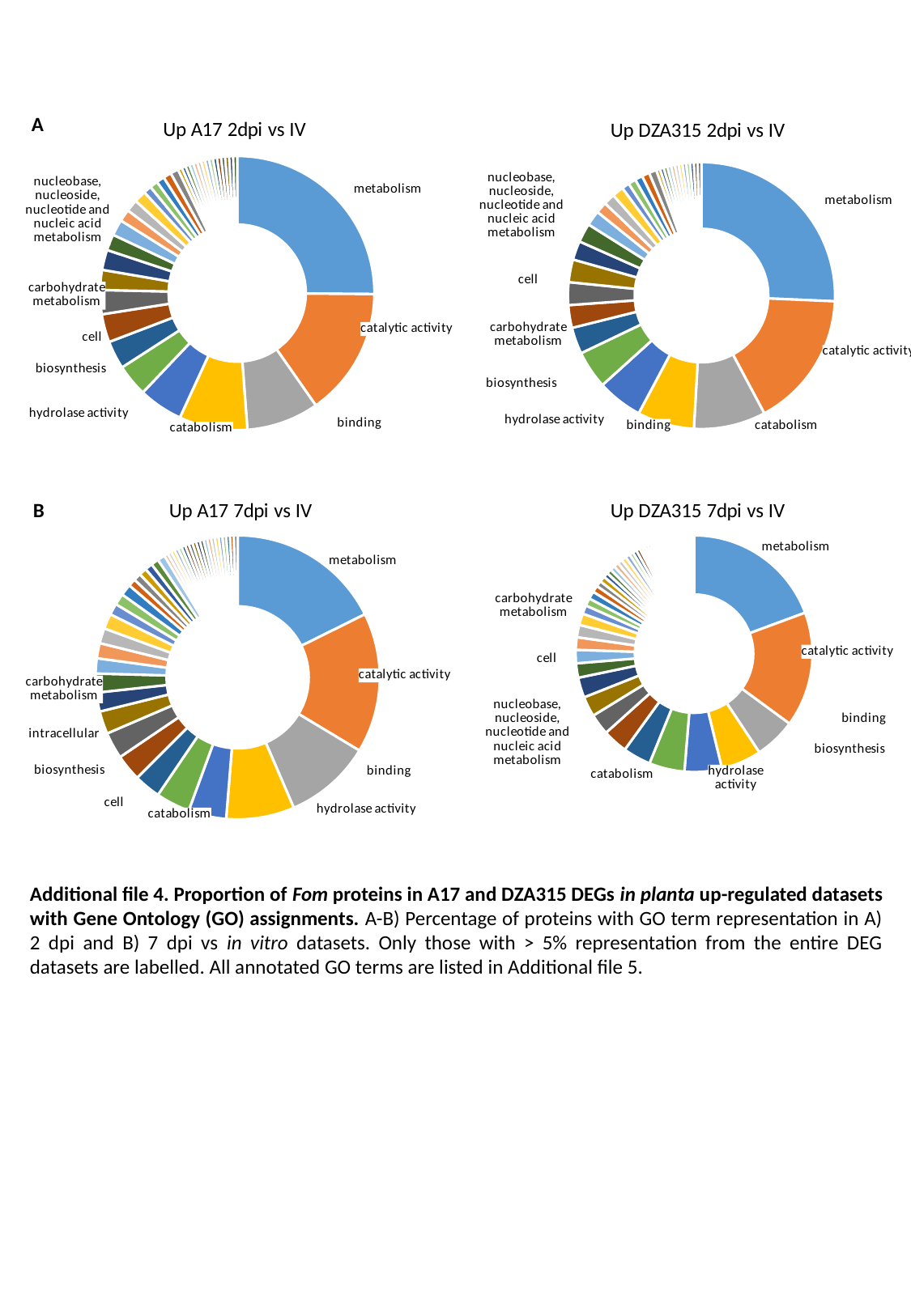

### Chart: Up A17 2dpi vs IV
| Category | % |
|---|---|
| metabolism | 49.532710280373834 |
| catalytic activity | 29.906542056074763 |
| binding | 16.822429906542055 |
| catabolism | 15.887850467289718 |
| hydrolase activity | 10.2803738317757 |
| biosynthesis | 7.476635514018691 |
| cell | 6.5420560747663545 |
| carbohydrate metabolism | 6.5420560747663545 |
| nucleobase, nucleoside, nucleotide and nucleic acid metabolism | 5.607476635514018 |
### Chart: Up DZA315 2dpi vs IV
| Category | % |
|---|---|
| metabolism | 47.863247863247864 |
| catalytic activity | 30.76923076923077 |
| catabolism | 16.23931623931624 |
| binding | 12.82051282051282 |
| hydrolase activity | 10.256410256410255 |
| biosynthesis | 8.547008547008547 |
| carbohydrate metabolism | 5.982905982905983 |
| cell | 5.128205128205128 |
| nucleobase, nucleoside, nucleotide and nucleic acid metabolism | 5.128205128205128 |
| transferase activity | 5.128205128205128 |
| peptidase activity | 4.273504273504273 |
| intracellular | 4.273504273504273 |
| lipid metabolism | 3.418803418803419 |
| cytoplasm | 2.564102564102564 |
| transporter activity | 2.564102564102564 |
| nucleotide binding | 2.564102564102564 |
| transcription factor activity | 1.7094017094017095 |
| protein metabolism | 1.7094017094017095 |
| DNA binding | 1.7094017094017095 |
| transport | 1.7094017094017095 |
| nucleic acid binding | 1.7094017094017095 |
| cell organization and biogenesis | 0.8547008547008548 |
| external encapsulating structure | 0.8547008547008548 |
| electron transport | 0.8547008547008548 |
| generation of precursor metabolites and energy | 0.8547008547008548 |
| development | 0.8547008547008548 |
| antioxidant activity | 0.8547008547008548 |
| cytosol | 0.8547008547008548 |
| extracellular region | 0.8547008547008548 |
| mitochondrion | 0.8547008547008548 |
| nucleus | 0.8547008547008548 |
| ion transport | 0.8547008547008548 |
| cell wall | 0.8547008547008548 |A
### Chart: Up A17 7dpi vs IV
| Category | % |
|---|---|
| metabolism | 32.03125 |
| catalytic activity | 28.90625 |
| binding | 17.96875 |
| hydrolase activity | 14.0625 |
| catabolism | 7.8125 |
| cell | 7.03125 |
| biosynthesis | 5.46875 |
| intracellular | 5.46875 |
| carbohydrate metabolism | 5.46875 |
### Chart: Up DZA315 7dpi vs IV
| Category | % |
|---|---|
| metabolism | 33.18385650224215 |
| catalytic activity | 26.905829596412556 |
| binding | 9.641255605381167 |
| biosynthesis | 9.417040358744394 |
| hydrolase activity | 8.74439461883408 |
| catabolism | 8.295964125560538 |
| nucleobase, nucleoside, nucleotide and nucleic acid metabolism | 6.502242152466367 |
| cell | 5.829596412556054 |
| carbohydrate metabolism | 4.932735426008969 |B
Additional file 4. Proportion of Fom proteins in A17 and DZA315 DEGs in planta up-regulated datasets with Gene Ontology (GO) assignments. A-B) Percentage of proteins with GO term representation in A) 2 dpi and B) 7 dpi vs in vitro datasets. Only those with > 5% representation from the entire DEG datasets are labelled. All annotated GO terms are listed in Additional file 5.
