# Supplementary material for: Transcriptome analysis of the fungal pathogen Fusarium oxysporum f. sp. medicaginis during colonisation of resistant and susceptible Medicago truncatula hosts identifies differential pathogenicity profiles and novel candidate effectors
Source: BMC Genomics. 2016 Nov 3;17:860. doi: 10.1186/s12864-016-3192-2 (PMC5094085; doi:10.1186/s12864-016-3192-2)
Supplement: Additional file 7: — Plots of DEG significance versus in planta up-regulated fold induction. (PPTX 77 kb) [file 12864_2016_3192_MOESM7_ESM.pptx]

## Slide 1
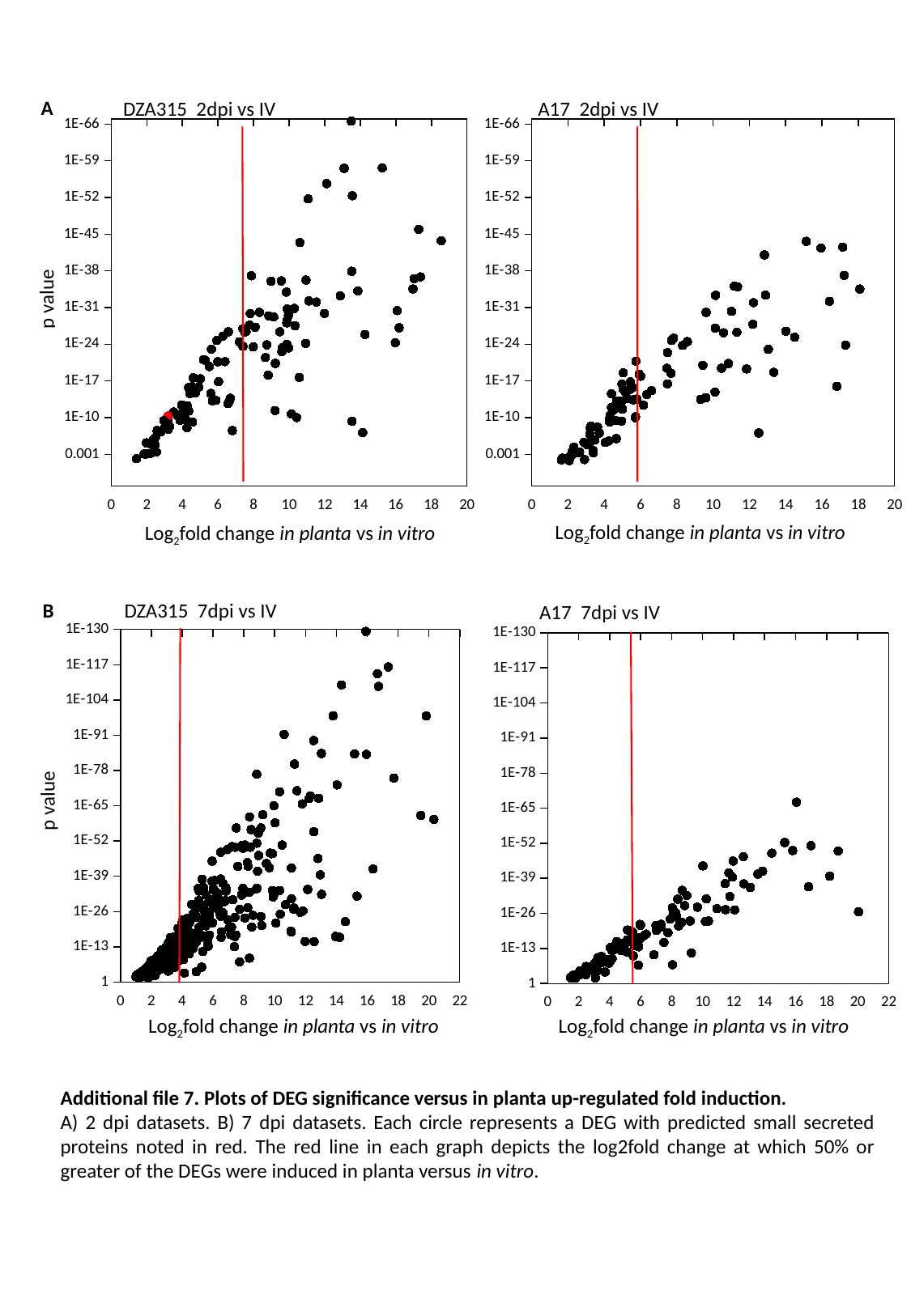

A
A17 2dpi vs IV
DZA315 2dpi vs IV
### Chart
| Category | pvalue |
|---|---|
### Chart
| Category | pvalue |
|---|---|p value
Log2fold change in planta vs in vitro
Log2fold change in planta vs in vitro
B
DZA315 7dpi vs IV
A17 7dpi vs IV
### Chart
| Category | pvalue |
|---|---|
### Chart
| Category | pvalue |
|---|---|p value
Log2fold change in planta vs in vitro
Log2fold change in planta vs in vitro
Additional file 7. Plots of DEG significance versus in planta up-regulated fold induction.
A) 2 dpi datasets. B) 7 dpi datasets. Each circle represents a DEG with predicted small secreted proteins noted in red. The red line in each graph depicts the log2fold change at which 50% or greater of the DEGs were induced in planta versus in vitro.
