# Supplementary material for: Transcriptome analysis of the fungal pathogen Fusarium oxysporum f. sp. medicaginis during colonisation of resistant and susceptible Medicago truncatula hosts identifies differential pathogenicity profiles and novel candidate effectors
Source: BMC Genomics. 2016 Nov 3;17:860. doi: 10.1186/s12864-016-3192-2 (PMC5094085; doi:10.1186/s12864-016-3192-2)
Supplement: Additional file 8: — qRT-PCR validation of candidate pathogenicity-associated genes in vitro versus in planta over an infection time-course. (PPTX 556 kb) [file 12864_2016_3192_MOESM8_ESM.pptx]

## Slide 1
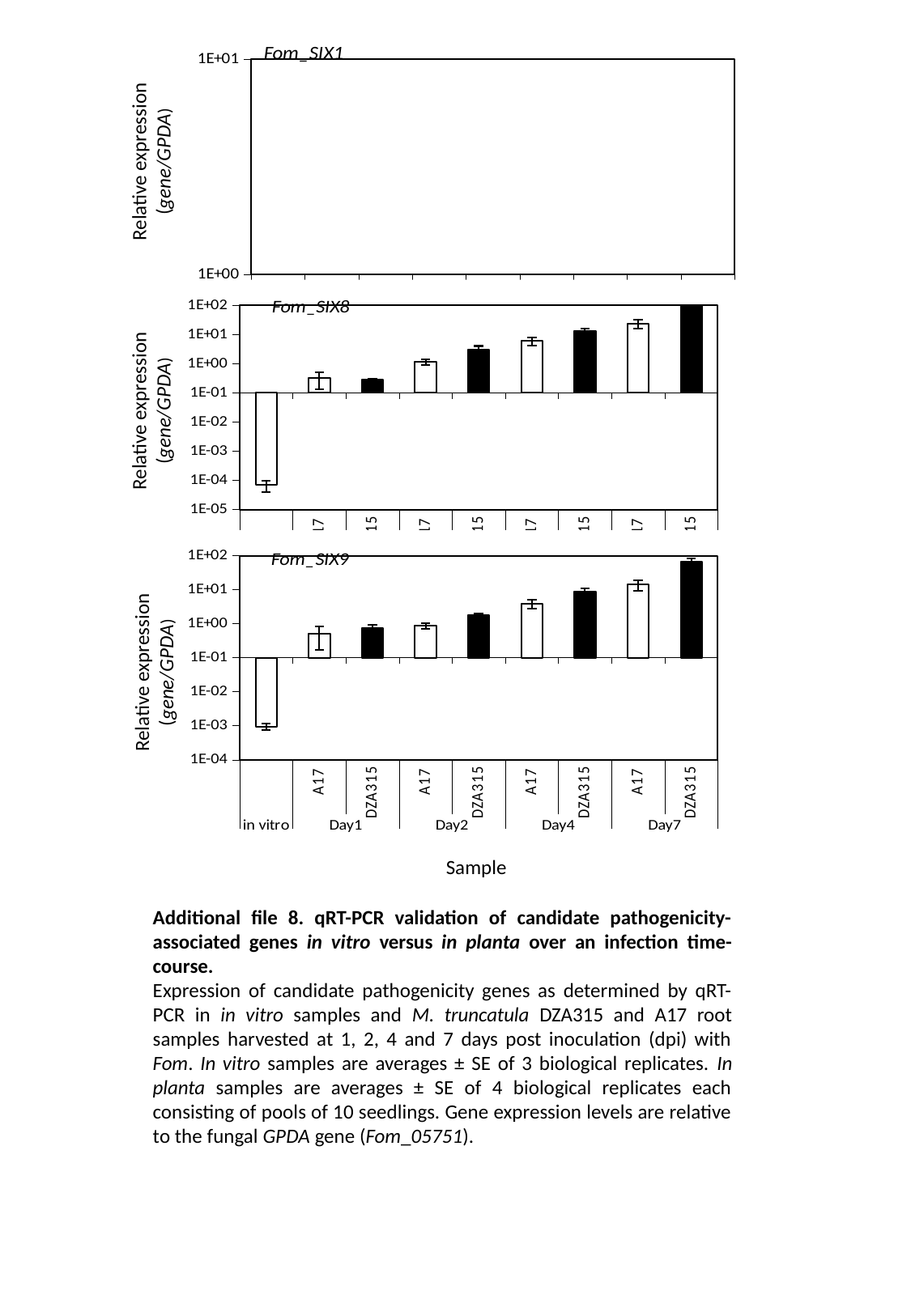

### Chart: Fom_SIX1
| Category | SIX1 |
|---|---|
| | 2.072130627176918e-05 |
| A17 | 0.0 |
| DZA315 | 0.0 |
| A17 | 0.0 |
| DZA315 | 0.0 |
| A17 | 0.02747222262480013 |
| DZA315 | 0.0322172985375981 |
| A17 | 0.16932444505385455 |
| DZA315 | 0.5204846688153141 |Relative expression (gene/GPDA)
### Chart: Fom_SIX8
| Category | SIX8 (LT) 328-329 |
|---|---|
| | 6.807746405419167e-05 |
| A17 | 0.31462172647805214 |
| DZA315 | 0.29012503674178 |
| A17 | 1.1579464961944057 |
| DZA315 | 2.981852844207173 |
| A17 | 5.911230235855114 |
| DZA315 | 13.129366203679265 |
| A17 | 23.55773606113378 |
| DZA315 | 94.65333583271905 |Relative expression (gene/GPDA)
### Chart: Fom_SIX9
| Category | SIX9 (LT) 481-482 |
|---|---|
| | 0.0009568582862724373 |
| A17 | 0.506030606489969 |
| DZA315 | 0.7280411326993551 |
| A17 | 0.8798388555462693 |
| DZA315 | 1.7488163852296068 |
| A17 | 3.866667083169433 |
| DZA315 | 8.843232913163451 |
| A17 | 14.004945942200754 |
| DZA315 | 65.58564552208209 |Relative expression (gene/GPDA)
Sample
Additional file 8. qRT-PCR validation of candidate pathogenicity-associated genes in vitro versus in planta over an infection time-course.
Expression of candidate pathogenicity genes as determined by qRT-PCR in in vitro samples and M. truncatula DZA315 and A17 root samples harvested at 1, 2, 4 and 7 days post inoculation (dpi) with Fom. In vitro samples are averages ± SE of 3 biological replicates. In planta samples are averages ± SE of 4 biological replicates each consisting of pools of 10 seedlings. Gene expression levels are relative to the fungal GPDA gene (Fom_05751).
